# Mental health of social media influencers

**DOI:** 10.1093/joccuh/uiae045

**Published:** 2024-08-14

**Authors:** Isabelle Bray, Moya Lerigo-Sampson, Yvette Morey, Joanne Williams

**Affiliations:** School of Health and Social Wellbeing, University of the West of England, Bristol, BS16 1QY, UK; Bristol Business School, University of the West of England, Bristol, BS16 1QY, UK; Bristol Business School, University of the West of England, Bristol, BS16 1QY, UK; School of Health and Social Wellbeing, University of the West of England, Bristol, BS16 1QY, UK

**Keywords:** social media influencers, mental health, wellbeing

## Abstract

There is a wide body of research on the effects of social media use on mental health, particularly focusing on young people. However, very little is known about the mental health of social media influencers (SMIs), who also tend to be quite young. This is of growing significance as more of our daily lives is conducted online, and in the context of poor population mental health globally, which declined further as a result of the COVID-19 pandemic. We therefore set out to review the mental health of SMIs and, in the absence of literature on SMIs, drew on other similar but more traditional occupational groups, such as the self-employed, to draw conclusions and identify directions for future research.

There has been a steady increase in social media use since the early 2000s.^1^^,^^2^ Research into the mental health effects of the use of social media, or “social networking sites” (SNSs), has grown exponentially since 2010. In 2020 two systematic reviews were published on the topic. The first reviewed 16 papers and found that half were cross-sectional. The authors reported that “although the results of the study were not completely consistent, this review found a general association between social media use and mental health issues.”^3^ They concluded that there was a need for more qualitative and longitudinal studies. The second also included 16 studies. Five of these reported positive effects, whereas 8 reported negative effects (3 were not categorized as either positive or negative). Although individual studies reported negative associations with a wide range of mental health outcomes including mood and self-esteem,^4^^,^^5^ SNS use was, overall, found to be related to less loneliness and greater self-esteem and life satisfaction.^6^ The authors reported that positive interactions, social support, and social connectedness on SNSs were related to lower levels of depression and anxiety, whereas negative interaction and social comparisons on SNSs were related to higher levels of depression and anxiety. This suggests that mixed findings regarding the relationship between social media use and well-being may be explained by different types of online interactions.^7^ Since different demographic groups use social media differently,^8^^,^^9^ it follows that certain groups are disproportionately affected.

Much of the concern about social media use and mental health has focused on the effects on young people, perhaps because they tend to be more avid consumers of social media than older generations,^8^ and because young people’s mental health has been a particular concern, even before the recent pandemic.^10^ Although all groups within society were affected in different ways by the COVID-19 pandemic and associated lockdowns, a systematic review found that those younger than 40 years were more severely affected.^11^ A review of COVID-19 and mental health outcomes in children and young people reported that it increased both depression and anxiety in adolescent cohorts.^12^ A review focusing on digital media use and mental health of adolescents during the pandemic reported that the negative aspects of use, such as social comparison and fear of missing out, were more likely to affect social media users during lockdown, due to social isolation and confinement.^13^ Most studies in this review reported a negative association between well-being and social media use.

A survey of social media users noted that the increasing necessity to use social media for work could lead to increased pressures on workers to be constantly “connected,” resulting in stress.^14^ This calls into question the well-being of people who use social media as an integral part of their working lives. Social media influencers (SMIs), or “content creators,” may be at an increased risk of the negative impacts on mental health because they are heavily immersed in social media as part of their job. SMIs are characterized by having sizable networks of active followers and are used to promote brands, products, or services through social media platforms such as Instagram, Facebook, X (formerly Twitter), and TikTok.^15^ An initial search of the literature has found no peer-reviewed evidence about the mental health of this occupational group, despite the fact that the numbers of people making their living in this way is growing rapidly,^16^^,^^17^ and accelerated during the COVID-19 pandemic, when reliance on the internet and social media for entertainment and information increased.^18^ Even before the pandemic, in 2018, 75% of advertisers reported using influencers and 43% expected to increase their spending on influencers in the next year.^19^ However, a parliamentary report on influencer culture identified “mental health issues from their constant online presence” as one of a number of challenges faced by this occupational group,^20^ highlighting the need for further research on this topic. The need to understand the well-being of SMIs is particularly important given that they tend to be young^21^ and female^22^ (compared with other occupational groups), both risk factors in themselves for poorer mental health.^23^^,^^24^ The sex difference in mental health morbidity becomes apparent during adolescence^25^ (attributed to factors associated with puberty such as body dissatisfaction and higher levels of upward comparison amongst girls^26^), and persists across the lifecourse.^27^ It is estimated that 84% of all SMIs are women, compared with just 16% men.^28^

In the absence of research evidence about the well-being of SMIs [based on a PubMed search using the terms “social media influencer*” AND (“mental health” OR wellbeing)], we have looked more broadly at the literature on those who are self-employed or freelance, SMIs being a subset of these categories [(“freelance*” OR “self-employed”) AND (“mental health” OR wellbeing)]. Since the longitudinal studies of UK civil servants first revealed that it was those with least control over their work who had the worst health outcomes,^29^ the finding has been confirmed in other studies,^30^ with pressure, stress, and autonomy potentially mediating the observed association with mental health. It would seem logical that the autonomy of being self-employed might yield well-being benefits for this occupational group. However, a systematic review of the literature^31^ found increased risks of a wide range of health-related outcomes—from sickness absence to suicide—for the self-employed, as well as some protective effects, such as higher levels of physical activity^32^ (likely to promote mental health^33^^,^^34^). The evidence in this review for other mental health outcomes was either inconclusive (depression) or showed no difference between the employed and self-employed (anxiety). Demographic factors appear to moderate the effect of being self-employed. For example, one study found that being self-employed was associated with depression in women but not men,^35^ whereas there is evidence that being self-employed is protective for older but not younger workers.^36^ As noted above, SMIs tend to be female and relatively young, again pointing toward an increased risk of poorer mental health outcomes for this occupational group. Before applying the systematic review findings to SMIs we should consider the quality of the evidence. Because 23 of the 26 included studies were cross-sectional, there is a possibility of reverse causality. In other words, it is possible that those with poorer mental health choose to be self-employed, as evidenced during the COVID-19 pandemic.^37^

It is also worth bearing in mind that since the pandemic, many employees have adopted some aspects of a lifestyle that previously were limited to the self-employed,^38^ giving more control over how and where work is carried out, potentially diminishing at least some of the differences between the self-employed and employees. However, it should be noted that employees tend to trade increasing flexibility for additional effort.^39^ It is widely accepted that the shift in working practices toward more home-working has brought benefits for older employees (such as more flexibility to accommodate caring responsibilities) but negative impacts for younger workers, who miss out on the informal mentoring and social interactions that the traditional workplace offers.^40–42^ This pattern mirrors the finding above in relation to the benefits of being self-employed for older but not younger workers.

Beyond the literature on self-employed workers, similar searches for research on health outcomes associated with contract [“non-standard employment” AND (“mental health” OR wellbeing)] or atypical working patters [“atypical worker*” AND (“mental health” OR wellbeing)] yielded no further relevant research. We conclude that there is a lack of evidence on the mental health and well-being of SMIs, and that this is an urgent area for future research. Authors of reviews of social media use and mental health have called for more qualitative and longitudinal studies^3^ and more randomized controlled trials.^6^ Because it is not possible to randomize people to the role of SMI, other epidemiological study designs, such as cohort and case–control studies, should be employed to provide a stronger evidence base examining the well-being of this occupational group than the broadly cross-sectional data that we have drawn on here. Qualitative work is also needed to understand the mechanisms by which the experiences of SMIs affect health outcomes, including coping strategies. The role of potential moderators, such as demographic variables and personality type, should also be investigated in future work.
